# Cellulose-Based Nanoparticles Processed from Agricultural Waste Biomass—A Review

**DOI:** 10.3390/nano16060387

**Published:** 2026-03-23

**Authors:** Shadrack Mubanga Chisenga, Francis Collins Muga, Olabisi Mariam Okesola, Jones Yengwe, Haibao Liu, Peter Kaluba, Alice Mutiti Mweetwa, Zizikazi Sodzidzi

**Affiliations:** 1 Department of Land Management, School of Agricultural Sciences, University of Zambia, Lusaka P.O. Box 32379, Zambia; jones.yengwe@unza.zm (J.Y.); peter.kaluba@unza.zm (P.K.); alice.mweetwa@unza.zm (A.M.M.); 2 Department of Agricultural and Rural Engineering, Faculty of Science Engineering and Agriculture, University of Venda, Thohoyandou 5050, South Africa; francis.onyando@univen.ac.za (F.C.M.); zizikazi.sodzidzi@univen.ac.za (Z.S.); 3 School of Engineering and Materials Science, Queen Mary University of London, London E1 4NS, UK; m.o.ashimi@qmul.ac.uk (O.M.O.); haibao.liu@qmul.ac.uk (H.L.)

**Keywords:** waste biomass, lignocellulose, cellulose, carbonized particles, nanocellulose, rheology, thermal decomposition, crystallinity, zeta potential

## Abstract

The nanoparticles processed from non-edible crop materials and residues have evoked great use in the food and non-food industry. The diversity in agricultural waste biomass and differences in extraction techniques account for variations in end-product properties, and would require examination of waste crop types (source) to determine suitability for the production of cellulose, nanocellulose and graphene particles. This review showed that screening criteria of end-user properties include chemical composition, cellulose contents, morphology, crystallinity, thermal stability, rheology, surface charge and zeta potential. The literature shows that the end-user properties vary with plant source (that is crop type) and extraction techniques. In this review, the cellulose content and percentage crystallinity are primary parameters for selecting agricultural waste biomass for the production of nanocellulose and nanofibrils. Additionally, zeta potential and surface charge can determine polymer interaction for suitability in industrial applications. Moreover, nanocellulose and biochar were found to have various industrial applications as ingredients in the production of food packaging including active packaging, rheological modifiers and thickeners. Pyrolysis is the eminent strategy for the transformation of agricultural waste into biochar-derived nanoparticles and carbon-rich materials.

## 1. Introduction

Agricultural waste is composed of non-edible and non-usable parts of food and non-food crops, including crop residues (straw, stalks, husks, leaves) and animal manure. The global production of major crops was estimated to produce over 16 billion tonnes of total biomass annually, of which ~80–85% are non-edible residues [1]. The objective for higher-yielding crop varieties would suggest an increasing amount of agricultural waste biomass. The landfilling biomass waste can lead to significant emissions of methane (CH_4_), a potent greenhouse gas contributing to climate damage and its consequent socioeconomic impact. In the US, municipal solid waste landfill tipping fees typically range from USD 50 to 100 per ton, whereas the private sector’s operational costs can exceed USD 1000 per ton due to specialized waste treatment and compliance requirements [2]. However, the cost of processing waste biomass into valued added products was approximately USD 300 per ton, a much lower cost than those of landfilling [3]. The global production of nanocellulose alone was estimated at 1000–2000 ton in 2024, and was projected to exceed 10,000 ton by 2030. The market size for nanocellulose was approximately USD 500–800 million in 2025, and is projected to reach USD 4 billion by 2035, with the broader biomass-derived nanoparticle markets growing at 10–15% Compound Annual Growth Rate (CAGR). The global leaders in the bio-based nanomaterial market are Canada, the US, Norway, and Japan. These countries exhibited advances in production technologies, and huge progress in research and development (R&D). The bio-based materials sector generated approximately 4–5 million jobs globally in biomass processing and R&D, with nanocellulose contributing close to 10,000–20,000 specialized jobs in 2024, specifically in production and application development. The bioeconomy was reported to increase global GDP by approximately 2–3% [4], with the nanomaterial sector performing positively through bio-alternative innovations and technology for the reduction of plastic waste costs by USD 100 billion annually.

Nonetheless, the increasing waste biomass has emerged as a concerning issue in many countries including South Africa and the broader Southern African region, where approximately 90% of general waste consists of biodegradable materials from food and non-food plant sources. The agricultural waste accounts for a large proportion of landfill materials and is prominently becoming a major environmental and economic concern. The concern of rising greenhouse gases was also ascribed to increasing agricultural waste [5]. Nonetheless, there has been a growing trend towards utilizing agricultural waste to develop value-added products based on biopolymers. Industrial efforts towards the valorization of agricultural waste have been centered on lignocellulosic biomass.

Lignocellulose biomass is a complex molecular structure, environmentally friendly and the most abundant renewable natural material derived from agricultural waste and forestry residues. Lignocellulose is a component of three major biopolymers of cellulose, hemicellulose, and lignin. The crude contents by weight of cellulose, hemicellulose, and lignin were reported in the range of ~35–55%, 20–40%, and 10–25%, respectively. The differences in composition and the content of these biopolymers can vary according to species, germplasm types and planting season [6] including methods of treatment and extraction techniques. The recalcitrance of lignin can limit the transformation of biomass into valuable products such as bioethanol. Lignin has a 3D structural network of phenylpropanoid units linked by ether and carbon–carbon bonds exhibiting a high molecular weight and density, and being of a hydrophobic nature. This structure complex was reported to hinder chemical or enzyme access to cellulose and hemicellulose, making it difficult to fractionate lignocellulose. In view of this, several extraction methods have been developed with a common goal of removing lignin to produce holocellulose (molecular structure of hemicellulose and cellulose together).

Cellulose is the major component of lignocellulose biomass, and can be extracted using alkaline and acid hydrolysis, and bleaching processes. Cellulose including raw lignocellulose materials can be pyrolyzed onto biochar, and subsequently graphitized into graphene particles, and can be activated using various processes such as acid and alkaline treatment including particle size reduction. Increasing demand for nanoscale sustainable materials and ingredients has elicited greater research interest in the production of nanomaterials from plant sources. Cellulose can be processed into nanocellulose and cellulose nanofibrils. This paper catalogs the chemical composition of raw agricultural waste biomass, processes for the extraction of cellulose, and the production of nanocellulose and biochar. Also, the paper interprets and explains the variations in nanocellulose properties such as surface charge, crystallinity, thermal stability, and zeta potential.

## 2. Composition and Extraction of Lignocelluloses

The composition of various agricultural waste materials is shown in Table 1. Agricultural waste biomass is a rich source of lignocellulose, a polysaccharide containing cellulose, hemicellulose and lignin.

### 2.1. Lignin Content

The highest lignin content (40%) was seen in olive leaf, making it a significant source of lignin, while olive wood and pruning parts yielded lower amounts of lignin. This is indicative of a variation in the lignin content across different parts of the same plant. Lignin is a phenolic polymer with an amorphous structure of three monomers, guaiacyl propane, syringyl propane, and 4-hydroxyphenylpropane [22], which are chemically cross-linked through alkyl–ether, carbon–carbon and aryl–ether bonds, and play a functional role in binding cellulose and hemicellulose into holocellulose. The organic solvent and alkali treatment processes are some of the common extraction methods for valorizing lignin into useful products. The reactive sites of the phenol ring interact with formaldehyde producing hydroxymethyl phenols linked through methylene bridges (C-C) and ether bridges (C-O-C) to form a cross-linked and rigid structural network. This mechanism signifies the role of pure lignin for industrial applications including the synthesis of emulsifiers, additives and phenolic resins. Yet there is limited scientific investigation on utilization of lignin in the production of sustainable ingredients. The higher removal rate of lignin from lignocellulose can result in increased concentrations of holocellulose (hemicellulose and cellulose).

### 2.2. Hemicellulose Content

Hemicellulose is a heterogeneous polymer of various monomers including pentoses and hexose sugars such as xylose, mannose, and glucose. Hemicellulose varies in the range of about 10–40% across different agricultural biomass. The xylan monomers are abundant in hardwood [23] and this can explain the higher hemicellulose content (40%) in rice straw than rice husk. In the plant cell wall, hemicellulose is bonded with cellulose fibrils through lignin cross-links of hydrogen bonds and van der Waals forces. Hemicellulose can be extracted or removed from the biomass through various hydrolysis methods using acid and alkaline treatment, and enzymatic processes. The hemicelluloses extracted from different biomass, including bamboo using the mild alkaline treatment, showed diverse monosaccharide compositions predominantly containing xylose [24]. Pure hemicellulose can alter its properties for specific applications such as increasing hydrophobicity and thermal stability through etherification, esterification, or cross-linking. These properties give hemicellulose a wide range of industrial applications including biodegradable films and coatings for food packaging.

### 2.3. Cellulose Content

Cellulose [(C_6_H_10_O_5_)n] is the most abundant and major polysaccharide of glucose in plants, serving a structural function rather than a nutritional role. It is an unbranched polymer (linear polysaccharide structure) of glucose residues joined by β(1-4)-glycoside linkages [25]. The structural (residue) of the chain is D-glucopyranosyl units. The β-configuration allows cellulose to form very long, straight chains of repeated units of D-glucopyranosyl units. The contents of cellulose vary in the range of about 20–85% across different agricultural waste biomass. The amount of cellulose by weight is greater than that of hemicellulose and lignin, making it the major component of lignocellulosic biomass. The differences in the crude content of cellulose were ascribed to germplasm of plant species, plant age, geographical origin, and season of planting [26]. The D-glucopyranosyl has hydroxyl functional groups responsible for hydrogen bonding within the same linear structure and with monomers of other different carbon chains. Cellulose fibrils can be formed when parallel chains interact through hydrogen bonding. This creates an entangled network of intramolecular and intermolecular hydrogen bonding into a tightly packed crystalline and fibrous structure. The straight chain formed by β linkages combined with structural entanglement can explain the high tensile strength in fibers. The cellulose fibrils were characterized to undergo thermal decomposition at high temperatures in the range of 300–315 °C. This thermal stability heightens the use of cellulose in the production of sustainable industrial lightweight products and nanoparticles [27]. The high-rate removal of lignin and hemicellulose proportionately increases the yield of cellulose. Cellulose can be extracted using conversional pulping methods including Kraft, sulfite and bleaching processes. Kraft pulping is an alkaline treatment, and in combination with a mixture of sodium hydroxide and sodium sulfide, the method yielded 72–85% of cellulose from sugarcane bagasse [28]. The sulfite method is acid hydrolysis, commonly using sulfuric acid and was reported to reduce 88% of lignin by volume while retaining 92% of extracted cellulose fibers [29]. Bleaching agents such as chlorine-based chemicals including hydrogen peroxide and sodium chlorite are added to the extracted cellulose for the purposes of increasing the whiteness index of cellulose. Additionally, bleaching agents have an additional role of degrading the amorphous region of cellulose [30,31]. It is worth noting that pure cellulose is extracted cellulose less its ash content. Please see the work of Zhang, M. et al. [32] on calculating the percentage cellulose adjusted against the content of its ash.

Cellulose can be classified into four allomorphs: cellulose types I, II, III, and IV. The differences lie in crystalline forms based on the arrangements of cellobiose units (D-glucopyranosyl) in the chain and hydrogen bonding patterns [33]. Cellulose type I is the native cellulose, mainly a combination of two co-existing crystal structures named I_α_ and I_β_, of which I_β_ is the major component in higher plants, and has a parallel packaging structure of hydrogen bonds [34]. Cellulose type II exhibited antiparallel packing of hydrogen-bonding due to chemical modification of native cellulose when treated in alkaline or acid hydrolysis. Cellulose type III is generated by treating native cellulose or cellulose II in liquid ammonia or other amines [35]. Cellulose type IV is generated using physical modification in which cellulose III or cellulose II is heated at temperatures of about 300 °C in plasticizing liquid medium such as glycerol. Cellulose IV showed high thermal stability at higher temperatures and this can explain its desired application in the production of high-grade cellulose fibers used in rayon manufacturing [36]. This showed that raw native cellulose can have limited applications, and would require that cellulose is functionalized using physical and chemical modifications. However, sustainable methods are desired in response to climate change objectives.

## 3. Green Methods for Extraction of Cellulose

**Figure 1:**
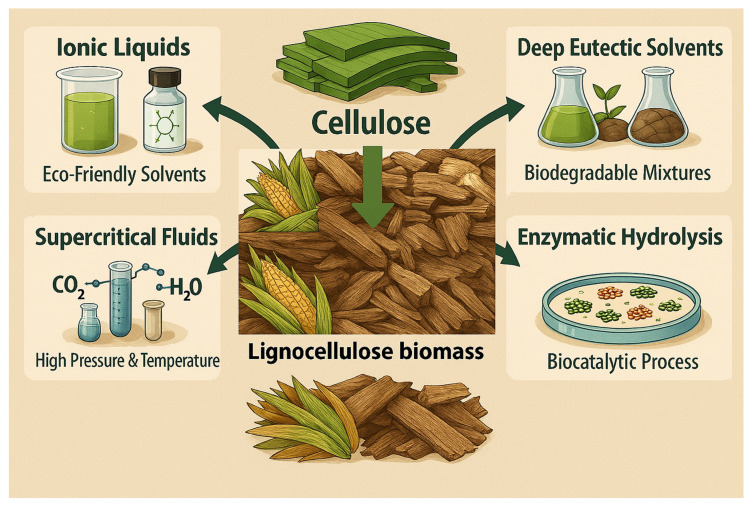
Schematic representation of green methods: ionic liquids, deep eutectic solvents, enzymes.

Green methods for cellulose extraction are aimed at environmentally friendly compounds and energy-efficient techniques. Although green methods are cost prohibitive and time consuming, they are reported to offer sustainable approaches for the extraction of cellulose from agricultural waste biomass. Some of the common sustainable methods include ionic liquid solvents and enzymes (Figure 1).

### 3.1. Ionic Liquid Solvents

Increasing environmental safety concerns due to the use of hazardous chemicals led to the development of green methods, some of which include ionic liquid solvents made of organic salts that are liquid at or near room temperature [37]. Imidazolium-based ionic liquids including 1-Dodecyl-3-methyl-imidazolium, 1-Decyl-3-methyl-imidazolium, tetra-butyl-phosphonium acetate and tri-butyl-methyl-phosphonium acetate were reported to dissolve corn stover, yielding a huge amount of cellulose in the range of 44–84% [27] under mild conditions of 80 °C, 3 h [27]. The cellulose-dissolving capacity of various ionic liquids are tabulated in the work of [38]. The factors influencing cellulose dissolution include viscosity, the degree of polymerization, ionic liquid hydrogen bond basicity, dissolution time and temperature. Ionic liquids with low viscosity have increased molecular mobility resulting in enhanced dissolution of cellulose. Nevertheless, the high viscosity of ionic liquids can limit the mass transfer rate resulting in decreased reaction and absorption rates [39], and attempts to increase pump force to aid mass transfer can be cost prohibitive. The melting point of ionic liquid combined with the thermal decomposition temperature of cellulose is the key criteria for selecting optimized dissolution temperatures. This is aimed at preventing pyrolysis from occurring, and thus avoiding thermal degradation of material. The ionic liquids of methylimidazolium and methylpyridinium with allyl-, ethyl-, or butyl- side chains were reported to yield better dissolution of cellulose. These side chain groups contain -OH, which enhances interaction with cellulose, and consequently, leads to better dissolution. The heteroatomic nature of side chains can give additional polarity. The double bond into the alkyl side chain of an ionic liquid reduces its viscosity compared to its saturated alkyl chain counterpart [40]. The high hydrogen-bond basicity anions such as Cl^−^ and CH_3_COO^−^ can yield a stronger hydrogen bond acceptor, which enhances disruption of inter- and intramolecular H-bond networks between cellulose molecules. The ionic liquid 1-ethyl-3-methylimidazolium acetate is commonly used because the CH_3_COO^−^ showed a high hydrogen-bonding basicity (>0.92) and molecularly dispersed solution of cellulose forms [41]. The challenges associated with ionic liquids include a high cost of production, and the lack of standardized regulations can limit the application of ionic liquids at scale.

### 3.2. Deep Eutectic Solvent

Extraction of cellulose using deep eutectic solvent is still research in progress, which began in 2012 by testing 26 different deed eutectic solvents on the dissolution of lignocellulose biomass [42,43]. Most of the listed deep eutectic solvents have shown limited dissolution capacity for cellulose. However, the deep eutectic solvent made of choline chloride and guaiacol in the 1:1 molar ratio was demonstrated to dissolve peanut shells, resulting in high yields of 60% cellulose with a reduction in lignin and hemicellulose at removal rates of 70% and 90%, respectively [14]. The hydrogen bond acceptor and hydrogen bond donor are key for the successful dissolution of cellulose. Acidic deep eutectic solvent DESs containing choline chloride in combination with lactic acid or oxalic acid are reported to be more effective at dissolving lignocellulose and removing lignin. The molar ratio of the H-acceptor to H-donor can significantly influence the viscosity and, consequently, extraction efficiency. High temperatures in the range of 100–140 °C decreases the viscosity of deep eutectic solvent, the mechanistic process that increases molecular mobility resulting in an enhanced mass transfer for improved penetration into the dense biomass structure. This demonstrates that moisture content and particle size can be manipulated for enhanced mass transfer. Assisted technology, such as microwave-assisted technology, can significantly accelerate the extraction process [43].

### 3.3. Enzymatic Process

The extraction of cellulose using enzymatic hydrolysis is commonly a multi-step process using ligninase enzymes such as laccase and lignin peroxidase to degrade lignin, and hemicellulose enzymes such as xylanase to break down hemicellulose. The digested lignin and hemicellulose can be separated from cellulose through filtration and washing [44]. The enzymatic system for cellulose extraction can be a blend of enzymes having different mechanisms of action that result in the degradation of lignin and hemicellulose, and retention of cellulose. The enzymes for cellulose extraction processes are microbial in nature, and commercially available. The ligninase (CAS: 80498-15-3) is the lactase responsible for catalyzing the oxidation of phenol-containing compounds through the reduction of oxygen to water [45]. The pectinase (CAS: 9032-75-1) has a functional role in breaking down the polysaccharide pectin [45], and aiding liquefaction processes by reducing viscosity for improved filtration and product clarity, and preventing particle sedimentation. The hemicellulase (CAS: 9025-56-3) is a glycoside hydrolase [45] with a functional role in hydrolyzing the bonds in heteropolysaccharides like xylan and mannan, and converting complex plant fibers into simpler sugars [46,47]. The enzymes of fungal species for aiding the production of cellulose include *Penicillium verruculosum*, *Trichoderma reesei*, *Aspergillus niger*, and *Sporotrichum Thermophile* [48]. However, the fibrous structural properties of various biomass feedstock are resistant to enzymatic hydrolysis. The interaction of lignin and hemicellulose through covalent/non-covalent bonds can form a rigid structure that limits enzymatic access to the lignin. To overcome these drawbacks in the prior addition of enzymes, the primary step is a pre-treatment process such as milling, boiling or heating aimed at degrading hemicellulose. Also, soaking in water can allow the material sample to imbibe water molecules, resulting in an expanded structure and thus increasing enzymatic accessibility. Additionally, soaking releases impurities which would otherwise hinder enzymatic performance. Most pre-treatment methods were selectively targeted at hemicellulose because of its lower degree of polymerization, make it an easy component to degrade. Yuan, X. et al. [49] demonstrated that the enzymatic hydrolysis of corn bract pre-treated in an ammonia peroxide mixture (NH_3_/H_2_O_2_) yielded a higher cellulose content (76%) compared to the cellulose content (52%) from glacial acetic acid (CH_3_COOH/H_2_O_2_) pre-treated corn bract (Table 1). Additionally, NH_3_-enzymatic hydrolysis showed higher removal rates of 86% and 70% for lignin and hemicellulose, respectively [49]. Ammonia carries amine functional groups that can disrupt the hydrogen bonds linking hemicellulose with other biomass components, resulting in increased accessibility for enzymatic action, leading to improved extraction of cellulose.

## 4. Production of Nanoparticles

**Figure 2:**
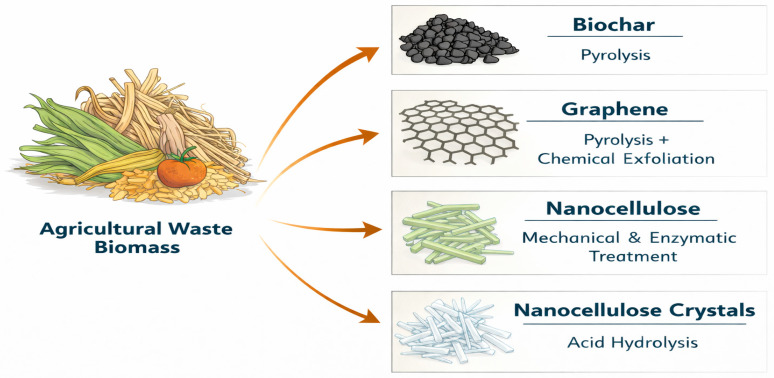
Nanoparticles processed from agricultural waste biomass.

Producing nanoparticles from agricultural waste biomass through carbonization processes represents a sustainable approach to valorizing abundant, low-cost residues like crop husks and peels into high-value nanomaterials. The nanocellulose is extracted from cellulose fibrils using various extraction methods. The major production methods of nanoparticles include carbonization, acid hydrolysis, enzymatic hydrolysis and the mechanical process (Figure 2).

### 4.1. Carbonization

Carbonization is the thermal decomposition of biomass in oxygen-limited conditions to yield carbon-rich structures at the nanoscale with reactive properties such as a high surface area and porosity. The techniques include pyrolysis, hydrothermal carbonization, and microwave-assisted carbonization. These processes are combined with activation steps for improved particle functionality. The raw waste biomass is prepared by screening and sorting to remove the foreign matter. The sorted biomass is washed using industrial water, with subsequent washing using deionized water, and drying at a temperature of 65–110 °C. Drying at low temperatures (45–65 °C) is aimed at maintaining the native biomatrix structure of lignocellulose. The dried biomass is ground into powder to reduce the particle size.

Pyrolysis is the common thermochemical technique of carbonization in which biomass is thermally decomposed into biochar when heated at a temperature range of 300–1300 °C under inert gas conditions in the furnace [50]. The resulting product is biochar, which is carbon-rich but mostly amorphous. The typical process utilizes carbon dioxide and a mixture of other inert gases including nitrogen, methane, and hydrogen. The choice of heating temperature is prescribed by the thermal properties of biomass. The common methods of pyrolysis are classified into slow, fast and gasification. Slow pyrolysis is characteristic of low heating rates (5–7 °C min^−1^) at a temperature range of 400–650 °C with higher residence times from hours to days [51]. This process can yield biochar 35–50% of biomass weight with a higher carbon content (70–80%). Fast pyrolysis operates in the temperature range of 800–1250 °C with heating rates (10–200 °C s^−1^), and short residence times (1–5 s), and can yield higher bio-oil (60–75%) than biochar (10–25%), and have a higher surface area [52]. However, fast pyrolysis is associated with biochar of a lower carbon stability due to rapid volatilization.

Steam pyrolysis is conducted using the biomass reactor for the production of various products including hydrogen, bio-oil and char [53]. The biomass reactor is heated to 100 °C followed by the injection of steam and nitrogen gas at a rate of 2 mL H_2_O_(g)_ min^−1^ and 30 mL min^−1^, respectively. The generated liquid products are collected in a glass liner located in a cold trap maintained at 0 °C. The liquid phase consisting of an aqueous and an oil phase can be fractionated to yield bio-oil. The steam pyrolysis of cotton seedcake using a Heinze reactor with a steam velocity 2.7 cm s^−1^ at various temperatures 400–700 °C yielded maximum char (27%) and oil (39%) at 400 °C and 550 °C, respectively. The low yields of biochar (17–22%) at higher temperatures (>400 °C) is indicative of increased secondary reactions as the pyrosis temperature increased. Nonetheless, the steam process can yield a natural porous structure in the biochar with a surface area as high as 1000 m^2^g^−1^ (Table 2).

### 4.2. Biochar Activation

The activation of biochar involves chemical treatments and physical methods, primarily drying and milling processes to form various allotropes of carbon including fullerene, graphene, carbon nanotubes, carbon nanofibers, and graphene-like nanosheets (Table 2). The activation is aimed at removing non-carbon components like O and H to yield a structure with improved porosity and surface area with increased carbon purity.

The common chemical agents are potassium hydroxide, potassium ferrate, metal catalysts such as iron, cobalt and nickel, and acids like sulfuric acid and phosphoric acid. The mixture of biochar and chemical agents are heated at various temperatures. Aro-Modiu, O. et al. [55] demonstrated the conversion of peanut shell carbonized powder to graphite by impregnating iron (III) chloride hexahydrate (FeCl_3_·6H_2_O) on carbonized powder, which was then pH-adjusted using hydrochloric acid. The mixture was stirred and heated at 60 °C for 5 h, and was left to undergo gradual evaporation for 7 days and final drying at 100 °C for 1 h using an air circulation oven. This process yielded 95% of graphene based on 2 g biochar [55]. Iron (Fe) acted as a catalyst by dissolving carbon and precipitating graphene structures. The chloride (Cl^−^) interacts with carbon and non-carbon components which increases surface area and porosity for enhanced adsorption capabilities [64]. The alkali [62]-activated biochar showed higher adsorption capacity by capturing environmental pollutants at a rate of 63–87 mg g^−1^ of nitric oxide compared to the native form of biochar at 18 mg g^−1^. Potassium interacts with the biochar structure between the carbon layers, creating forces that disperse the layers apart, leading to the formation of tiny spaces (pores) and thus increasing porosity; and the OH^−^ group introduced more oxygen-containing functional groups and thus improved surface chemistry. Anthonysamy, S. et al. [57] demonstrated that increasing the amount of alkali impregnation on a unit biochar weight basis from 1:1 to 3:1 increased the surface area of graphene from 390 to 712 m^2^ g^−1^, the positive change in surface chemistry of which can be ascribed to enhanced interactions between oxygen and carbon, and this can increase the formation of the carboxyl group, hydroxyl group and epoxy groups.

Acid activation can impact on the physicochemical properties of biochar by creating acid functional groups on the biochar. The acid hydrolysis is aimed at removing the metallic impurities, and introduces new functional groups in the inner pores and on the surface; it builds negatively charged surface areas, and thus modifies the physicochemical properties of biochar [65]. Some of the commonly utilized acidic agents include sulfuric acid, hydrochloric acid, nitric acid, oxalic acid, phosphorous acid and citric acid [66]. However, the environmental safety concerns led to growing interest in green chemistry principles in reference to principle 3—less hazardous synthesis [67]. In view of this, organic acids such as carboxylic acid (-COOH) can be used to functionalize biochar. Some of the common organic acids containing -COOH functional groups are acetic acid, citric acid, tartaric acid, oxalic acid and malic acid [68]. Carboxylic functionalized biochar can find various applications including catalysis and biochar-supported nanostructures.

### 4.3. Production of Nanocellulose

The extracted cellulose finds application as feedstock in the production of nanoparticles of a biological nature derived from natural fibers. The nanocellulose materials are classified as nanofibers, nanocrystal, nanofibrillated, and nanocrystalline (Table 2). The other category is bacterial cellulose nanocrystal; however, the present work focused on nanomaterials of a plant origin. The nanomaterials can be differentiated based on source and morphology (size, shape, form) including aspect ratio. Both nanofibers and nanocrystals processed are from cellulose and exhibited at least one dimension in the range of 1–100 nm; however, nanofibers showed a higher aspect ratio than nanocrystals. The nanofibrillated materials are composed of cellulose nanofibers in the form of a gel or film. Similarly, nanocrystalline materials are composed of cellulose nanocrystals in the form of a gel, film, or membrane. Milling is the size reduction process and the key activity in the production of nanoparticles and the activation process. The milling process-assisted technologies included ultrasound of ultrasonication, high-pressure homogenization, and microwave [69]. Other mill process-assisted techniques included hydrolysis of an acidic, alkaline, water or alcohol nature. Organic solvent-assisted ball milling can be used in the production of nanocellulose.

### 4.4. Mechanical Milling

Ball milling is the mechanical process of grinding materials into the finest powders at the nanoscale. The mechanical method uses high shear forces in the range of 20–30 kWh kg^−1^ to break down fibers into fibrils (Table 3). The equipment is primarily made of a cylindrical jar packed with balls. The balls are made of durable materials such as steel or ceramic. The cylindrical jars are designed to rotate. The milling machine with stationary housing (cylindrical jars), with an impeller attachment of a rotating arm inside the cylindrical jar, are commercially known as an attritor mill or stirred ball mill. The rotating arm or jar causes motion among the balls, resulting in the shearing or colliding of the balls against each other and the housing walls, and this action releases energy required to transform the particles from a micro size to the nanoscale. This mechanism, in the presence of the sample material, causes impact, compression and shear forces not only for particle size reduction, but also for mixing and blending the materials. This mechanical milling as a material processing technology for nanomaterials has wider applications in various industrial areas including pharmaceutical, bioengineering, and packaging. The technology does not require the use of strong chemical agents, and has been described as an environmentally safe method. Nonetheless, there is a serious challenge relating to the reproducibility of nanomaterials at scale, specifically difficulties in predicting milling conditions and suitability for material refinement. Additionally, high times, high energy requirements or low sample quantities per process including contamination, amorphization and the stability of nanoparticles are significant challenges limiting the commercialization of laboratory experiments for the large-scale production of nanopowders. The decrease in crystallinity and undesirable change in crystalline lattice were common challenges associated with ball-milled cellulose. In view of this, much of the recent authored works focused on process optimization in the function of independent variables (number and size of balls, time, rotational speed and weight of material). Other variables included the status of milling (dry or wet), the weight ratio between balls and the material sample.

The types of ball millers commonly used in the industry, and in laboratories, include the planetary ball mill, mixer ball mill, and vibration ball mill [77]. The planetary ball mill has been extensively reported for processes of the defibrillation of cellulose and lignocellulose biomass. The common speed of ball milling was reported in the range of 200–700 rpm and time range of 0.5–20 h as tabulated in Table 3. The higher speed can accelerate size reduction and fibrillation compared to lower speeds. Naghdi, M. et al. [70] studied the characteristics of biochar-derived nanoparticles after ball milling, using a designed experiment based on variation in the key factors of time, rotational speed and ball-to-powder-mass ratio. The result showed that the combination of a higher rotational speed and short time (575 rpm, 1.6 h) generated the finest particles at 212 nm compared to powder with 453 nm particles obtained from an experiment with a lower rotor speed and high time (540 rpm, 7 h). The high rotation speed can generate high collision energy resulting in reduced milling time. However, dry milling, combined with high friction energy, can cause overheating, which can damage microfibrils. To prevent overheating, the rationing of machine power over milling times was demonstrated [70] by setting the machine on and off at time intervals of 5 min to facilitate heat dissipation. Other works reported a resting phase of 10 min in cycles of 20 min of milling for the rotor speed of 400 rpm for 3 h, and this implied a minimum of five resting phases in 3 h [74]. The resting period minimizes degradation of the crystalline region of cellulose that could possibly occur with continuous milling. The alkali and bleaching pre-treatments of cellulose, combined with alkaline-assisted milling, yielded cellulose nanofibrils with a higher crystallinity and lower crystal size D-values of 3 nm than those processed from water pre-treatment dry ball milling [74]. Phanthong, P. et al. [71] investigated the properties of cellulose after ball milling with different time treatments (0.5, 1, 2, and 3 h) at 300 rpm. The result showed that an increase in ball milling time decreased the crystallinity and reduced the crystal size of ball-milled cellulose. The crystallinity index decreases exponentially with time due to disrupted hydrogen bonds and polymorph transformation from the native cellulose allomorph type I to cellulose type II. The decreased crystallinity can be associated with the increase in the amorphous region, and the yield of nanocellulose increased with milling time. The milling time is the driving factor for structural disruption and size reduction, and can lead to agglomeration. The decreased crystallinity, increased amorphous region, and consequent agglomeration were commonly associated with dry milling. It is worth noting that the mechanical milling process is not selective because it impacts both crystalline and amorphous structures, causing some damage to the crystalline domain. The effect of wet milling using solvents in a ball miller aimed at minimizing the loss of the crystalline region was investigated. Dos Santos, D.F. et al. [76] ascribed the increase in crystallinity to the combination of ethanol-assisted wet milling, longer hours, and low speed. This optimal condition resulted in better fibrillation. Ethanol can cause a swelling of the ground matrix, making the structure accessible and accelerating removal of the amorphous region and water, including non-cellulose components such as lipids. The ethanol–cellulose interaction is based on hydroxyl groups (OH^−^) forming hydrogen bonds between molecules, and this action can cause the precipitation of nanocellulose.

### 4.5. Aggregation of Nanoparticles

The aggregation of particles during synthesis can influence dispersion, stability, and the end-user properties of nanoparticles. The clustering of nanoparticles is driven by intermolecular forces and can lead to bundles, dimers, or larger aggregates [78]. The mechanical fibrillation including grinding and ultrasonication can increase the surface area and -OH groups leading to agglomeration. The drying methods such as spray-drying can increase hydrogen–hydrogen (H-H) interactions by promoting the formation of hydrogen bonds. The evaporative effect can concentrate the suspensions by the removal of water leading to increased inter-particle attractions and entanglement among the particles. The aggregation can be identified or recognized when the particle size exceeds their native morphological structures. Using Atomic Force Microscopy, the cellulose nanocrystals were observed to aggregate into clusters with a size exceeding the mean values of the length (180 nm) and height (6 nm) of the native nanocellulose crystals [79]. This aggregation was associated with a lack of sonication in particle suspension and centrifugation. The aggregation of particles was shown to be common in the hybridized nanoparticles of a catalytic or magnetic nature. The nanocellulose impregnated with inorganic nanoparticles (NiO) yielded a high surface energy, leading to induced clustering [80]. In hybrids, the ionic strength, surface charge and pH are some of the factors influencing the aggregation of particles. The metal ion concentrations can induce electrostatic repulsions between charged particles; in particular, sulfate groups from sulfuric acid hydrolysis can lead to aggregation [81]. The adjustment of pH in aqueous media is key to altering the zeta potential and stabilizing suspensions and dispersions. In non-polar solvents, the -OH groups can heighten hydrogen bonding in between the same polymers, leading to self-association. The low surface charge of native nanoparticles can lead to flocculation. The strategies to stabilize suspensions and prevent aggregation are a function of the surface charge, mechanical processing, salt (ion) concentration, and drying method.

The surface functionalization of nanocellulose particles using TEMPO (tetramethylpiperidine-oxyl radical)-mediated oxidation processes can introduce -COOH groups, which can increase negative charge and electrostatic repulsion, thus reducing aggregation. This strategy is aimed at raising the surface charge from low to high. Alternative methods to alter the polarity of suspensions and prevent re-aggregation during drying include silylation [62]. The lyophilization of suspensions can minimize hydrogen bonding, which can result in the production of individualized (non-aggregated) nanoparticle powders [62,82]. Using a low ionic concentration can reverse aggregation [83]. The mechanical processes that integrate assisted technologies, such as sonication and ultrasonication, combined with chemical treatment can limit excessive fibrillation which might otherwise increase -OH exposure. The key parameters for sonication processes include sonication time, amplitude and power, energy input, and concentration of the suspensions [84].

### 4.6. Surface-Based Cellulose–Polymer/Water Interaction

The interaction of nanoparticles including nanocellulose with cellulose nanocrystals, cellulose, lignin, and hemicellulose are driven by hydrogen bonding and van der Waals forces, and influenced by surface chemistry, moisture (water), and polymer structure. The interaction of cellulose nanocrystals (CNC) with different hemicellulose compounds (glucomannan and xylan) was demonstrated in an experiment on functionalizing Atomic Force Microscopy levers with different percentages of CNC coverage using the Langmuir–Blodgett technique [79]. The interaction, expressed as adhesion force (nN), was described as a response factor in the function of the percentage CNC-functionalized lever coverage. The result showed that the interaction behavior of CNC-glucomannan and CNC-xylan were significantly similar, exhibiting increasing adhesion forces with increasing CNC-composite coverage. However, the CNC-glucomannan interactions significantly showed higher adhesion forces (6–100 nN) than those of CNC-xylan (5–20 nN) [79]. The differences in adhesion forces can be ascribed to structural differences between glucomannan and xylan, the major polymers of hemicellulose. The distinct difference in structural properties can yield significant adhesion and binding properties. Both polymers are linear polysaccharides with a β-(1→4)-glycosidic linked backbone with different sugar structures whereas glucomannan is a hexose (C6), and xylan a pentose (C5). The level of chain branching and substitution is higher in xylan than glucomannan, and this property strongly influences the interactions with water and polymers [85]. The low chain substitution level in glucomannan enables closer alignment and effective deposition on cellulose nanocrystal and nanofibrils. Composites made from glucomannan exhibited a nanolayered structure and strong intermolecular hydrogen bonds resulting in a higher tensile strength and great biocompatibility [86]. This can explain why glucomannan is a highly applicable ingredient in the production of fibrous materials requiring viscosity, gelation and adhesion such as thickeners and binders.

Cellulose–cellulose interactions increases hydrophobicity, the tendency of macroscopic cellulose materials to exhibit insolubility in water [85]. The molecular structure of cellulose is amphiphilic in nature with its glucose rings having polar -OH groups and non-polar C-H groups distributed along the glucose ring structure. The elimination of water through a heat treatment process can strengthen cellulose–cellulose interactions via intermolecular hydrogen bonding between -OH groups (polar–polar) of the adjacent fibrils, and hydrophobic interactions between C-H groups (non-polar), leading to an irreversible and densely packed aggregate of fibrils. The result of such fibrous structures exhibited reduced water-holding capacity, limited flexibility, and higher brittleness. The removal of lignin and hemicellulose causes pore closure in the cellulose matrices, and this can increase the tendency to aggregate. Industrial fibrous matrices with a good amount of residual lignin and hemicellulose are less prone to irreversible aggregation. Lignin and hemicellulose act as material spacers in cellulose-based composites, and thus limit the direct cellulose–cellulose interaction [87]. Water-resistant cellulose fibers are fabricated by decreasing the amount of hemicellulose. Conversely, an increased coverage of hemicellulose in cellulose–water systems can improve the reswelling (swelling) of fibrils. Less crystalline and disordered hemicellulose or holocellulose has a high water diffusion coefficient, and this can improve water accessibility to the cellulose chain in contact points of cellulose–hemicellulose. Wet heating (temperature), when combined with a specific heating time factor, induces the mobility of bound water within the cellulose fibril structure. The kinetic energy causes disruption and the rearrangement of molecular hydrogen bonds with increased diffusion of water, creating a disordered region and expansion in-between microfibrils, which give an impression of swelling in cellulose. It is worth noting that fibrils do not swell; instead, the highly hydrophilic cellulose fibrils and cellulose aggregates develop repulsive osmotic pressure when hydrated cellulose surfaces are in contact with water. The inclusion of hemicellulose simply facilitates the separation of microfibrils from one another [85]. The expanded cellulose structure increases the exposure of -OH groups and this can enhance microfibrils’ interaction with other polymers like starches, including plasticizers such as glycerol and sorbitol. The wettability of cellulose can be improved by altering surface charge density and chemical composition. Ion-rich cellulose, specifically carboxylated charged cellulose nanofibrils with counterions (Li^+^, Na^+^, Mg^+2^), was reported to yield a higher swelling capacity than those of carboxymethylated cellulose and acid-catalyzed cellulose. The interaction of water with cellulose in acid-catalyzed hydrolysis involves the breaking of β-1,4-glycosidic bonds connecting the glucose units, and this can reduce osmotic pressure, resulting in a low swelling capacity. The monovalent ions (Li^+^, Na^+^) can yield significant electrostatic repulsion, in part due to dissociated carboxylate salts and excessive ions. The carbonyl group (RCOO^−^), rich in electrons, binds with cations through electrostatic (ion–dipole) interactions to neutralize the charge. When negatively charged adsorption sites (RCOO^−^) are saturated, the excessive cations in water repel against each other, leading to the formation of electrostatic double-layer repulsion between similarly charged sites. This explains the highly expanded networks and maximum swelling of fibrils in cellulose–water systems. Generally, in nanocellulose–water interaction, the reaction mechanism for nanocellulose with water is essentially similar to interactions in cellulose–water systems. The difference, however, lies in that the high surface-area-to-volume ratio of nanocellulose due to high exposure of -OH groups can yield significant changes in kinetics and surface interactions.

## 5. Characterization of Nanoparticles

Particle size, dimensions, and shape are some of the factors that can influence the end-user properties of nanocellulose such as the surface charge, crystallinity, and thermal stability.

### 5.1. Surface Charge

The zeta potential (ζ-potential), expressed in modulus, is a surface charge and a key primary parameter that measures the electric charge on the surface of nanoparticles when dispersed in aqueous medium, and in the most part controls rheological behavior. The surface charge can be ascribed to protonation and deprotonation on the particle surface, and the formation of an electric double layer [88]. It is an indicator of electrostatic repulsion between particles, useful for predicting the stability of nanoparticles and colloidal behavior. The zeta potential of nanoparticles from cellulose were reported in the range of −16 mV to −60 mV [70,72,75]. The stability behavior of nanocellulose in suspensions can be classified as stable for modulus ζ > 30 mV. In this range, surface charge prevents aggregation of the particles. The suspensions with modulus ζ < 15 mV are described as highly unstable and can exhibit rapid coagulation. The suspensions ζ ~20 mV can show initial stability with gradual aggregation of particles. Other reports showed that nanoparticles with a ζ between −10 and +10 mV are considered neutral. The differences in the reported data on zeta potential can be ascribed to particle size. The smaller particles are likely to register higher surface charge density and thus higher modulus zeta potential [89]. The aqueous solution with higher electrostatic repulsion showed less aggregation and greater colloidal stability [90], and was dependent on the particle size, composition and pH of the medium [91]. Zeta potential is pH-dependent and can be controlled using electrolytes, surfactants, and surface chemistry modifications. Native nanocellulose has lower |ζ| values, and may require modifications or the inclusion of additives for stability of dispersions and suspensions. The TEMPO-oxidized nanocellulose achieves very stable suspensions (−50 mV to −98 mV), mainly ascribed to the high negative charge due to increased COO^−^ groups. The cellulose nanocrystals hydrolyzed and activated in sulfuric acid are more negative (~−50 mV) due to an increase in ${SO}_{4}^{-2}$ groups. The development and modification of nanomaterials are aimed at colloidal stability by avoiding aggregation of particles and achieving high modulus values for zeta potential. The zeta potential can be measured using Zetasizer Nano-ZS (Malvern Panalytical Ltd., Malvern, UK), a common instrument based on principles of electrophoretic mobility of particles [72]. For computing ζ, the velocity at which particles move toward a positive electrode is measured 15–16 times repetitions at a specified time such as 10 s for each trial [70,72,75].

### 5.2. Particle Size and Shape

Based on the size and dimension of the particle, the classification of nanomaterials is tabulated in Table 4. The dimensions are confined to the nanoscale size in the range of 1–100 nm in which the nanomaterials are classified into four categories: zero-dimensional, one-dimensional, two-dimensional, and three-dimensional, abbreviated as 0D, 1D, 2D, and 3D, respectively. The 0D has three dimensions (length, width, height) within the nanoscale, along with aspect ratio (L/W). When one dimension is >100 nm, the particle is referred to as a 1D nanomaterial. The 2D nanomaterial has one dimension <100 nm, while the other two dimensions are >100 nm. The 3D particle has three dimensions >100 nm. In this measurement, the thickness or thinness of the particle is considered as the diameter. There is no known nanocellulose material that was reported to exist in the range of zero-dimensional materials. The nanocellulose particle sizes were reported in the range of 2–450 nm. Although reported data described particles >100 nm as nanoparticles, it is worth noting that nanoparticles should fall in the category <100 nm. Nanocrystals and nanofibrils exhibited particles less than 4 and 30 nm, respectively, and different morphologies. The cellulose nanocrystal hydrolyzed using sulfuric acid have the amorphous region removed, resulting in a more crystalline particle with narrow size distributions compared to nanofibrils. Transmission electron microscopy showed that nanofibers were fibrils, filamentous-like in shape, while nanocrystals showed rigid and rod-like structures. The variations in shape and size can be ascribed to differences in ball milling speed and time. The longer milling time led to insignificant differences in particle size, and yielded the lowest nanocrystal size [92]. The ball milling-assisted processes, including wet milling of both water and chemical agents, and ultrasonication, yield smaller nanoscale particles than dry milling and unassisted milling [93]. The particle size distribution and average particle size at the nanoscale can be analyzed using Zetasizer Nano S90 apparatus (Malvern Instruments, Malvern, UK), which operates based on the laser beam scattering technique. The cellulose nanocrystals (5–20 nm) at 1 wt% improved the tensile strength of the composite by 82%, yielding 32 MPa [94]. The inclusion of cellulose nanofiber in polyhydroxybutyrate nanocomposites increased elongation at the break [95]. Nanocellulose particles have a high aspect ratio which enhances mass transfer networks and stress transfer, improving tensile strength and modulus. The nanofibrils are longer in length compared to nanocrystals, and this can enable nanofibrils to facilitate entanglements, along with elongation with an enhanced toughness of the composite.

### 5.3. Surface Area

The catalyzing effect of nanomaterials lies in the function of surface area. The surface area of nanomaterials from waste biomass were shown in the range of 110–2060 m^2^ g^−1^ (Table 2). The lowest and highest surface area were reported in nanocellulose and graphene, respectively. The variation in surface area could be ascribed to differences in activation processes. During pyrolysis, the lignocellulose components (hemicellulose, cellulose, and lignin) are thermally decomposed, and volatiles (gas, tar) escape leading to the formation of micropores in the resulting product of solid carbon. Du Plessis, M. [100] demonstrated that surface area was inversely related to the pore diameter of nanomaterial; and porosities of <50 μm formed interconnected biomaterials. The micropores (<2 nm) and mesopores (2–50 nm) can contribute significantly to the total surface area of bionanomaterials. The ball milling of solid carbon (biochar) can create micropores (<2 nm) within the carbon matrix as graphene-like sheets are formed and rearranged. The thermal decomposition temperatures can reorganize the carbon atoms into disordered nanocarbons (graphene-like sheets), leading to an increased surface area. The source and composition of waste biomass can influence the surface area of nanomaterials. The pyrolysis of wood materials with higher lignocellulosic contents were shown to produce nanocarbons with a higher surface area. The presence of silica and alkali salts in grasses and straws can limit the surface area by promoting particle aggregation through the formation of Si-O-Si bonds [101]. Similarly, materials with a high ash content such as manure were associated with a low surface area. A high ash content can promote particle size agglomeration [102], and signified ash as a parameter for the selection of cellulose for suitability in producing nanoparticles. A higher percentage of ash in biomass would suggest less cellulose content. Microfibrillation significantly increases the specific surface area of nanocellulose. The combination of ball milling with assisted technology such as high-pressure homogenization can break down the cellulose fibers, resulting in individual nanofibers or nanofibril, which are flexible and can form a highly entangled network with many exposed surfaces [103]. The oxidation of nanofibrils can increase the surface area. The 2,2,6,6-tetramethylpiperidine1-oxyl (TEMPO)-oxidized cellulose nanofibrils exhibited an increase in surface area. The TEMPO oxidation process introduces negatively charged carboxylate groups on the surface of the cellulose microfibrils. The gas sorption analysis using nitrogen as a carrier (Brunauer–Emmett–Teller Method) to generate N_2_ adsorption/desorption isotherms is the common method of measuring the surface area of dry nanocellulose powders [104]. Other methods include the open-cell tetrakaidekahedron method, specifically measuring the surface area of bacterial nanocellulose. In this method, the biological activity of a scaffold depends on its surface area per unit volume, and the specific surface area was associated with pore size and relative density [105].

### 5.4. Morphologies of Nanocellulose

The different types of nanocellulose such as nanofibrils and nanocrystals can exhibit a variation in morphologies (size and shape) based on the origin of the nanomaterial, extraction method, and activation processes. The acid hydrolysis can break down and remove the amorphous region of cellulose to generate cellulose nanocrystals with rod-like and whisker-shaped, and rigid structures. The removal of the amorphous region increases crystallinity [106]. The rod-like shapes can measure 3–50 nm in diameter, and have a length of 50–500 nm with an aspect ratio of approximately 5–50. However, the treatment time and acid concentration can influence the size of particles. Rana, M.S. et al. [107] demonstrated that an increased concentration and reaction time of sulfuric acid on cellulose resulted in particle size reduction from 800 nm to 600 nm, at 20 min and 45 min treatment time, respectively. Nevertheless, increased reaction time decreased the yield of cellulose nanocrystals by approximately 10%. The exposure to high concentrations of acid over longer times can degrade the amorphous region, and this probably explains the resulting smaller grain length of cellulose nanocrystals. The cellulose nanofibrils showed a long fibrillar network, and flexible and entangled web-like structure. The mechanical processes combined with assisted technologies, including high-pressure homogenization and pre-treatment of enzymatic or chemical nature, are a series of processes for the transformation of cellulose into cellulose nanofibrils with intact crystalline and amorphous regions [108]. The individualized cellulose nanofibrils exhibited a diameter of 3–5 nm and a length of 100–2000 nm. The surface topography, including shape and size, can be examined and measured using scanning electron microscopy [101], Atomic Force Microscopy (AFM) and Transmission electron microscopy [109,110]. These techniques are limited in measuring the length of highly entangled networks of nanofibrils and non-spherical nanomaterials. To overcome this challenge, the microscopy techniques are integrated or combined with other software techniques to facilitate the measurement of length, diameter or width. The microscopy image outputs can be processed using ImageJ software [111] or DiameterJ software [112] to determine the size of nanocellulose particles [113]. The sample suspensions of nanofibers and nanocrystals were examined using dynamic light scattering (DLS) and field-emission scanning electron microscopy (FE-SEM) [114]. The DLS can measure the diameter of spherical nanoparticles with the same diffusion coefficient in water. Thus, in the work of Tarrés, Q. et al. [114], DSL was applied to estimate the hydrodynamic diameter of rod-like cellulose nanoparticles for assays where the scatter angle and the consistency were uniform. Using a forward scattering angle, and varying the concentrations, are some of the recommendations for assays with different scatter angles. The FE-SEM determined the diameter of the nanofiber bundles, and the length of fibrils was estimated using geometrical relations [114]. The sedimentation and agglomeration of particles hinder the measurement of dimensions, and such limitations can be addressed by increasing polydispersity using appropriate concentrations within the range of 0.05–0.75 wt%. Depending on the analytical technique, concentrations of >1wt% are possible. The combinations of DSL with field-emission scanning electron microscopy and Transmission electron microscopy (FE-SEM and TEM) were used on colloidal solution of 2 wt% nanocellulose [72].

### 5.5. Crystallinity

The molecular structure of elemental cellulose contains fibril units bound together through hydrogen bonds between the OH^−^ groups of the anhydroglucose molecules in the chain. These fibril units comprise two structural domains, which are crystalline and amorphous. The crystalline domain is a well-ordered molecular structure while the amorphous is structurally disordered [115]. The elemental fibrils can bundle together through hydrogen bonding to form a nanostructure called microfibrils, also known as nanocellulose (or nanofibrous material). The hydrogen bonding can exhibit specific geometric arrangements of atoms through covalent bonds and electrostatic attractions, creating a different structure configuration and molecular orientation, often linear but also complex networks like rings or chains (dimers, trimers) in liquids and solids. Accordingly, the variation in configuration can yield different material properties. Based on the hydrogen configuration, molecular packing and orientation, cellulose is classified into different crystalline structures called polymorphs (I, II, III, IV) [116]. The polymorphs are characterized using unit cell lattice parameters of the six fundamental dimensions (three edge lengths, a, b, and c, and three interaxial angles, α, β, and γ) that are used to define the size, shape, and orientation of the smallest repeating unit (fibril) in a crystal structure, allowing the construction of the entire crystal lattice [117]. The orientation or chain configuration can be described as parallel or antiparallel in the function of edge lengths and interaxial angles. Cellulose I, a naturally occurring crystalline structure of fibrous material, was reported to have two substructures, namely cellulose I_α_ and I_β_ [117]. These polymorphs are described as parallel chain configurations based on I_α_ lattice parameters of length a (0.672 nm), b (0.596 nm) and c (1.040 nm) and interaxial angles α (118.08°), β (114.80°) and γ (80.38°) while the I_β_ lattice parameters were of length a (0.778 nm), b (0.820 nm) and c (1.038 nm), and interaxial angles of α (90°), β (90°) and γ (96.50°). The variations in chain configurations can be assigned to differences in the source of cellulose, extraction method and treatment methods.

The relative amount of crystalline and amorphous in a given cellulose sample can be described by the crystallinity index. X-ray diffraction (XRD) is the common technique of measuring the degree of the crystallinity index. The common operation in most XRD techniques is the Cu Kα (Copper K-alpha) radiation with characteristic X-ray wavelength (around 1.54 Å or 0.154 nm) and can be generated using a voltage of 40 kV and amperes of 40 mA (Table 5) at angular positions of 2θ (2theta) in the range of 2–70°. The data output from XRD is X-ray diffraction spectra, and it can be analyzed using the Segal method to calculate the percentage crystallinity index [117,118]. The XRD spectra can be used as criteria for the selection of fiber materials for suitability in processing nanocellulose. The percentage crystallinity of cellulose was reported in the range of 20–88%, and varied according to chemical composition, extraction method and treatments. The XRD spectra for raw rice husk and keya leaf showed several XRD peaks at various 2θ positions (Table 5). The peaks are indicative of the distinct lattice planes of the fibrous structure, and spectra with several peaks are a sign of cellulose I, a native fibrous material rich in lignocellulose contents. The chemical treatments such as alkalinization and the bleaching of cellulose I (i.e., raw agricultural waste) can degrade the amorphous region and subsequent removal of hemicellulose and lignin, and thus these treatments eliminate other peaks, resulting in a single peak and a rise in the crystallinity index. This progressive elimination of peaks can be a quality criterion for the successful production of nanocellulose and nanocellulose crystals. The source of cellulose can influence the crystallinity index. The crystallinity index (40–56%) of raw rice husk varied according to the rice variety [119]. This suggested that specific sources of plant can determine the genetic structure of the cellulose, including the ratio of its crystalline to amorphous regions. Nonetheless, there are limited studies that demonstrated the crystallinity index as a function of genetic factors such as the plant germplasm of the waste biomass. The variation in the crystallinity index according to the XRD technique and operation inputs were shown to be insignificant (Table 5). This implied that different XRD techniques and specific operational procedures such as sample preparation or data analysis methods like the Sego method yielded consistent and non-varying results.

### 5.6. Thermal Properties

The thermal stability is the most desired thermal characteristic, an indicator of the temperature at which the cellulose and nanocellulose material can thermally decompose. In most of the literature, the thermal decomposition temperature of nanocellulose was reported in the range of 150–350 °C (Table 6). Some of the factors that can explain the variation are the source of the nanomaterials, the extraction method, and the surface chemistry including contents of lignin and hemicellulose. The molecular crystal structure of nanofibrous material can also influence the process of the thermal decomposition of the cellulose. The nanocrystals derived from the process of acid hydrolysis have the amorphous parts of the cellulose degraded by the acid molecules; on the other hand, the crystalline region of the cellulose is resistant to the acid and remains undegraded. The high crystallinity index (80–90%) of cellulose nanocrystals was shown to correlate positively with higher thermal stability and decomposition temperatures [124]. Thermogravimetric analysis (TGA) is a common non-destructive technique for generating thermal profiles on the thermal properties, structure and performance of cellulose and derived cellulose materials intended for possible high-temperatures applications. In the TGA method, the samples are heated in the temperature range of 20–600 °C at a constant heating rate of 20 °C min^−1^ under nitrogen gas flow using an instrument STA 6000 (PerkinElmer, Waltham, MA, USA). The generated graphs can be analyzed using software packages such as OriginPro software [125]. The thermogravimetric graphs are plotted as weight% in the function of heating temperatures. Hoseinpour, Z. et al. [75] showed that derivative thermogravimetry data exhibited three-stage thermal curves of nanocellulose processed from cumin husk. The first curve was associated with an endothermic peak temperature required for initial weight loss as a result of drying due to the evaporation of bound water molecules and volatile compounds. This stage has a slower degradation rate due to the higher thermal resistance of more-ordered cellulose components. The second curve showed the onset degradation temperature for the degradation of hemicellulose and breakdown of some parts of amorphous cellulose. In this stage, the peak degradation temperature (T_max_) is indicative of the depolymerization of cellulose into volatile products, primarily anhydro-saccharides such as levoglucosan, and the initial formation of char, gases (carbon dioxide and water vapor), and volatile organics. The third stage was associated with the thermal decomposition of the remaining parts of the amorphous and crystalline cellulose, leading to volatilization and the complete formation of char structures. The thermal decomposition of fiber and nanocellulose derived from bamboo exhibited similar weight loss profiles at the initial drying stage and peak decomposition temperatures [126]. However, the final decomposition showed higher weight loss of up to 15 wt% in nanocellulose than those in fiber (12 wt%). The nanocellulose exhibited higher residue weight loss and lower decomposition temperature than those of cellulose [73]. The surface area can explain these observations. The nanocellulose has a higher surface area with less impurities, and hence lower resistance against heat transfer. The high surface area was negatively correlated with thermal stability, suggesting that as the surface area increases, the nanomaterial becomes highly susceptible to thermal decomposition [127]. Although nanocellulose has higher crystallinity than cellulose, the factors associated with the “nano” scale and extraction process can contribute to the reduction in its thermal stability. The process of producing cellulose nanocrystals using sulfuric acid hydrolysis can modify the particles by introducing sulfate groups on the surface. The interaction of sulfate groups with hydroxyls can release water molecules. This water can lower both the onset temperatures and activation energies of cellulose pyrolysis. The acid hydrolysis can enhance the breakdown of the β(1→4) glycosidic bonds of the cellulose chains, leading to an increased production of reducing ends or terminal ends of cellulose. This can lead to a decreased degree of polymerization. The decreased degree of polymerization and increased number of reducing ends can account for the lowering of the onset temperature. The sulfate increases the formation of charred residue, which acts as flame retardant, and this can also decrease the onset temperature.

## 6. Rheology Behavior of Nanocellulose

The study of the rheology properties of nanocellulose are aimed at understanding the impact of force on the flow and deformation behavior of nanocellulose in dispersions and suspensions. The flow or deformation behavior can technologically define the suitability of nanocellulose for processing. In practice, rheology relates to mixing properties of nanocellulose when included as an additive (nanofiller) and ingredient in suspensions (fluids). The suspensions are plasticizers such as water, glycerol and sorbitol [128], to mention just a few common examples. The role of a plasticizer is to raise plasticity, workability, and swelling capacity through the disruption of intermolecular forces, and to generate mobility in the polymer chains [129,130]. The disruptor is the applied force or torque (shear stress). The nanocellulose and cellulose suspensions were reported to exhibit non-Newtonian fluids, expressing properties like high viscosity and gel-like structures at low shear rates. The viscous or gel-like structure can be dominated with the formation of a structural network through hydrogen bonding, entanglement and the electrostatic interactions of chains. Depending on the rheological behavior, cellulose, nanocellulose and nanofibrils can find industrial application in coatings, food additives, cosmetics, 3D printing, and as rheology modifiers [131]. The network of nanocellulose can exhibit solid-like gel or a flocculated structure, as well as behavior characteristics of yield stress, high viscosity and viscoelastic at resting state (without external force). The yield stress is associated with nanocellulose behaving like a solid, sustaining its shape until the external force (stress) is greater than the specific threshold. The viscoelasticity behavior relates to elastic properties, soft solid or gel, with the ability to recover after small deformations. The external force, when applied, can break down the network (solid-like gel) of nanocellulose into shear-thinning, the state of deformation and flowing like liquid [131]. The external force generates shear stress due to the stirring, shaking, or pumping of cellulose or nanocellulose suspensions [132].

The nanocellulose, cellulose nanofibrils and cellulose nanocrystals act as nanofillers, influencing the mechanical properties of biocomposites made from biodegradable polymer matrices such as starch. The biocomposites can be processed using melt compounding, extrusion, injection molding, solvent casting, and 3D printing [133], and these processes are governed by rheology (mixing properties). In view of this, rheology properties find use in process control and systems analysis, and the design of piping technology.

**Figure 3:**
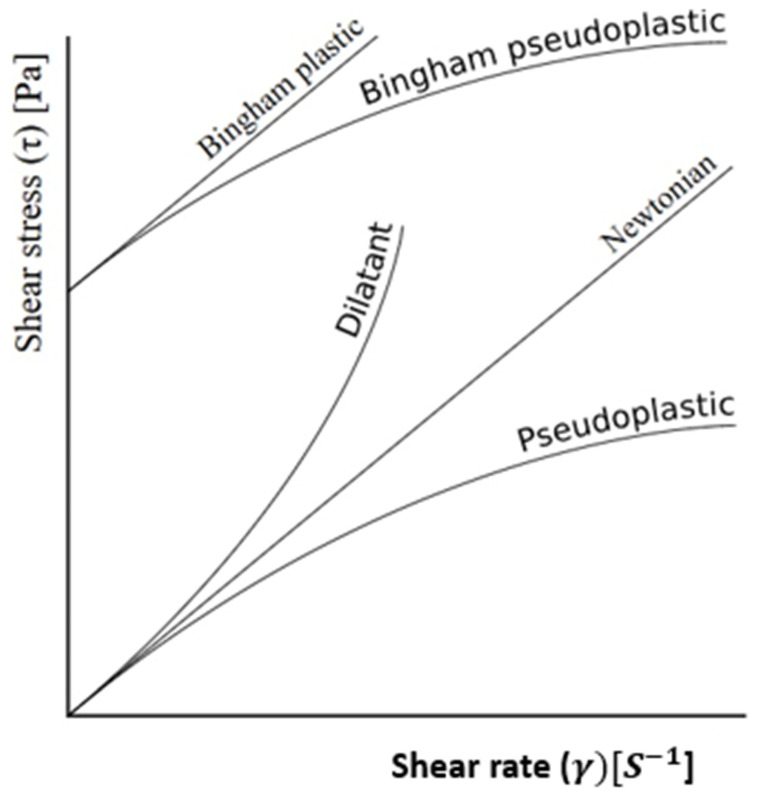
Example of stress–strain curves for non-Newtonian dispersions or suspensions.

The interaction between nanocellulose and starch or other base matrix polymers can increase viscosity by developing a network of hydrogen bonding and structural entanglement. This interaction can limit polymer chain mobility, resulting in elevated viscosity. The addition of 1–5 wt% cellulose nanocrystals into thermoplastic starch decreased the melt flow index from 2.10 g/10 min to 1.40 g/10 min [134]. Similarly, the inclusion of cellulose nanocrystals into polylactic acid film composite suspensions resulted in increased viscosity [135]. This suggested increased molecular weight retention, indicative of an increased formation of hydrogen bonding between ester and hydroxyl groups, and entanglement in coated samples. The cellulose nanocrystals have a high aspect ratio and rigid structure which creates chain entanglement, resulting in increased hydrodynamic volume, setting up resistance against the flow of composite suspension [136,137]. The percentage inclusion of cellulose nanocrystals, >3–5% by weight, was reported to build agglomeration in the suspension and can negatively influence the mechanical properties of polylactic. Such nanocellulose dispersions exhibited minimum yield stress to initiate the flow. This shows that below minimum stress, the composite remains in the state of a gel (soft solid behavior). In fact, this is a vital behavior for stabilizing dispersions or suspensions of a gel nature in paints and in the food industry. The application of force greater than stress yield can cause internal structure deformation, resulting in increased molecular mobility, leading to the flow of the suspension. This effect is called shear-thinning (pseudoplasticity), and is the desired state for casting, spraying and melt flow, and compression processes. The rheology properties can be characterized using torque rheometers [138] to generate shear stress–shear rate (γ°) curves or viscosity (η)–shear rate (γ°) curves. Rheological properties such as the storage modulus (G′), loss modulus (G″) and complex viscosity (η) are key parameters for describing the flowability and flow nature of the composites. The G′ and G″ are measured when strain (shear rate) is applied [139]. The flow behaviors are characterized using rheology models such as Herschel–Bulkley [140,141], Power Law [142] and Bingham Plastic [141]. The curves (Figure 3) can describe the nature of the flow of the polymeric composite solution. The gel structure with G′ > G″ is indicative of a solid-dominant material. Both G′ and G″ correlated positively with the increasing loading of cellulose nanocrystals into nanocellulose composites [135]. This is indicative of the composite exhibiting increasing stiffness and viscosity with higher energy dissipation. This behavior is a good example of dilatant fluids (Figure 1) showing shear thickening properties such as an increase in viscosity with the rate of applied shear stress—for example, the behavior of starch in water. The pseudoplastic (thinning) properties were illustrated in [139].

## 7. Application of Cellulose-Derived Nanoparticles

Nanoparticles such as nanocellulose, nanocrystals and biochar particles can be used as ingredients, additives and nanofillers in the production of biocomposites. The functionality of nanoparticles in biocomposites is in the function of thermal stability, capacity to form H-, C-C bonding and entanglement with polymer matrices. Additionally, rheology is the key quality criteria for deciding the usefulness of nanoparticles in viscous flow technologies.

### 7.1. Packaging

Nanocellulose serves as an additive or ingredient in the production of high-barrier packaging films. Poly-(butylene succinate), a biodegradable polymer matrix, was coated and laminated using the layered spray-coating technique at ~0.4% nanofibrillated cellulose of the biocomposite weight [143]. The resulting film decreased the water vapor transmission rate by approximately six-fold. Additionally, optical transparency of the laminates showed a significant drop from 70 to 30% of the visible light spectrum [143]. The nanocellulose can create excellent oxygen barriers, protecting food from oxidation. The mixture of polyvinyl alcohol/cellulose nanofibrils supplemented with iron ions yielded a composite film that showed a decrease in the oxygen transmission rate of approximately 70% [144,145]. Iron (Fe^3+^) enhanced bonding by improving cross-linking and the interaction of carboxyl with hydroxyl groups. Edible coatings loaded with nanocellulose yielded an antibacterial surface on strawberries, resulting in extended shelf life [146,147]. The high surface area and charge of nanocellulose heightens the interaction of nanocellulose with the bacterial cell membrane, increasing interference with DNA/proteins, which can probably disrupt bacterial biofilms. Nanocellulose can act as carrier of antibacterial agents.

The cellulose nanocrystal was mixed with silver nanoparticles to prepare the nanocomposite ingredient for the production and fabrication of antibacterial paper [148]. Nanocellulose can find application in the processing of foams and cushioning products. The cellulose nanofibrils facilitated the production of ultra-lightweight aerogels and foam products with improved ductility and thermal insulation with great thickness recovery [149]. The nanostructured, salinized cellulose nanofibrous frameworks generated a 3D nanoporous structure and exhibited silica aerogel materials of a lightweight nature with am insulating capacity of ultralow thermal conductivity of around 15.9 mW m^−1^ K^−1^ [149]. The biochar impregnated with silver nanoparticles (ZnO, Zn) showed suitability for development of active packaging materials by exhibiting an excellent combination framework of good electrical conductivity (6.7 × 10^−4^ S m^−1^), higher antioxidant potential with an inhibition capacity of >8 h, and higher antibacterial activity against bacterial pathogens [150,151].

### 7.2. Rheology Modifiers and Thickeners

The cellulose nanofibrils can form pseudoplastic fluid, a shear-thinning gel in water at low concentrations of the range of 0.1–2 wt%. This implies that under shear-thinning, the cellulose nanofibrils can quickly cause a decrease in the viscosity of gels, and can rapidly restore gel viscosity when stress is released [152]. This rheological property makes cellulose nanofibrils a good candidate ingredient in the food industry for the production of stabilizers for application in sauces, creams, ice cream, and whipped toppings, replacing fats or synthetic thickeners. The cellulose nanofibrils were used as an aqueous phase in the preparation of oil-in-water Pickering emulsions, the process of which resulted in an emulsion with increased zeta potential and viscosity, indicative of improved stability in the emulsions [153]. The emulsion instability associated with the use of native nanocellulose was addressed by preparing cellulose nanofibrils with a high carboxyl content using sodium periodate and a TEMPO two-step oxidation process to produce Pickering emulsions using the assisted technologies of ultrasonic emulsification and high-pressure homogenization. The resulting fabricated carboxyl-cellulose nanofibrils showed excellent stability with a creaming index of the emulsion of more than three months under storage conditions of a low temperature, 4 °C [154]. The inclusion of cellulose and cellulose nanofibers into shear stiffening gel resulted in a composite structural framework of cellulose-supported stiffening gel and CNF-supported stiffening gel, with a peak storage modulus of 908 MPa and 15.3 MPa, respectively. These properties inhibited the cold flow deformation of stiffening gel and showed enhanced impact-resistant performance [155]. Nanocellulose derived from *Ulva lactuca* (sea lettuce) was blended with fluoride and tested for antibacterial activity against *Streptococcus mutans* and *Lactobacillus acidophilus* [156]. The resulting composite exhibited higher inhibition capacity against the pathogens, making it an antimicrobial agent for application in tooth paste [156].

### 7.3. Biomedical Application

Cellulose-based nanoparticles including cellulose nanocrystals (CNC) and cellulose nanofibrils (CNF) are applied in biomedicine, mainly in the form of modified nanoparticles with a high degree of biocompatibility. The target properties are a high surface area to facilitate effective drug loading and enzyme immobilization. High exposure of hydroxyl groups on cellulose allows for the functionalization of surface chemistry using TEMPO-oxidation and cationization. Some of the key examples of applications include drug delivery, wound healing, tissue engineering, and diagnostics.

#### 7.3.1. Drug Delivery

The nanocellulose particles have been used as a carrier of doxorubicin for the treatment of human lung adenocarcinoma (A549 cells) with indicative pH-dependent performance of 97% drug release over 34 days [157]. The cationic-modified CNCs are binders of hydrophobic drugs such as docetaxel and paclitaxel [158]. This delivery system is reported to have enhanced solubility and controlled release. The hybridized composite CNC/alginate nanoparticle was developed to carry rifampicin (a potent antibiotic) for controlled minimal release at a gastric pH and high release at an intestinal pH (~7.4) [159]. This delivery system is almost insoluble in acidic conditions. The CNC-based hydrogels find application in contact lenses for the delivery of lysozyme into the eye.

#### 7.3.2. Tissue Engineering

The CNF processed from pineapple leaves combined with polyurethane produced superior properties for the production of high-tensile-strength nanocomposites for heart valve and cardiovascular replacements [160]. The conductive neural scaffolds made from nanocomposites of polypyrrole-coated cellulose acetate are used in electrical stimulation to guide neural cell growth [161].

#### 7.3.3. Other Biomedical Uses

Negatively charged, electrospun cellulose acetate nanofibers modified with dendritic structures have been developed to efficiently capture circulating cancer cells (KB cells) for diagnostic purposes. The high selectivity and water permeability properties of cellulose acetate make it suitable for use as a membrane material for hemodialysis.

### 7.4. Energy Sector

The progress of research on paper-based lithium-ion batteries showed the development of integrated Li-ion batteries on cellulose paper [162,163]. In these studies, carbon nanotubes and lithium titanium oxide were coated onto paper, with the cellulose acting as a conductive scaffold, separator, and mechanical support for wearable electronics. The carboxymethyl cellulose can enhance the cycle life of silicon-based anodes and is used as an eco-friendly binder, replacing non-biodegradable polyvinylidene difluoride [164].

The PANI-modified cellulose is a conductive composite produced by polymerizing aniline on nanocellulose substrates using chemical oxidative polymerization. The PANI-modified cellulose was used to develop high-performance supercapacitors characterized by flexible electrodes of high specific capacitance (656 F/g) and excellent cycling stability [165].

The cellulose nanofibril paper with a higher transparency and high haze, with better light-trapping properties, is suitably applied as a substrate for the production of organic solar cells with a high power conversion efficiency. The CNF networks are used to develop CNF-templated photoelectrodes, a mesoporous network template with high porosity and improved electron transport properties for dye-sensitized solar cells. There is progress in research into the development of cellulose-based nanogenerators for mechanical energy harvesting. Some of the nanogenerators include triboelectric nanogenerators and piezoelectric nanogenerators. The hybrid cellulose nanofiber aerogel-based triboelectric has been designed to generate a high voltage of 170 V and power (352 µW) to run light emitting diodes [166]. Sulfonated cellulose nanofiber membranes are developed as an alternative to Nafion in proton-exchange membrane fuel cells, and can attain a power density of 156 mW cm^−2^ [167].

## 8. Potential Harmful Effect of Nanoparticles

The potential beneficial applications of nanoparticles (1–100 nm) are clear in many industrial fields including cosmetics, packaging, electronics, and environmental remediation. Nonetheless, their nanoscale size (<100 nm) combined with a high surface-area-to-volume ratio, and superior physicochemical properties, can lead to potential harmful effects and toxicity in humans through inhalation, ingestion, dermal or intravenous. The nanoparticles may contain inorganic materials that possess health concerns, such as silver, titanium dioxide, zinc oxide, silica, and carbon nanotubes. However, there is limited information on the chemical composition of nanoparticles derived from lignocellulose. In fact, contamination with heavy metals, specifically lead, cadmium, arsenic and mercury, were reported in edible crops when they are grown in polluted soil, irrigated with wastewater, or exposed to industrial emissions [168]. This can indicate that the agricultural waste of such crops is likely to produce tainted lignocellulose-based nanoparticles. The cytotoxicity in vitro study investigated the effect of cellulose, nanocellulose, lignin-containing nanocellulose and lignin on the proliferation of human cells [169]. The result showed that cellulose (0.1–0.2%) had an insignificant effect on cell viability, while media containing lignin exhibited significant toxicity at low concentrations (0.02–0.1%) yielding 90%. The lignin-containing nanocellulose showed modest cytotoxicity at the concentration range of 0.02–0.05%. However, increased concentrations (0.1–0.2% lignin-containing nanocellulose) led to a reduction in cell viability by 60–85%. In additional experiments, lignin was shown to inhibit the growth of cancer cells (H460 and HEK293), while higher concentrations (0.2–0.4%) of nanocellulose showed insignificant cytotoxicity against HEK293 and H460 cells [169]. The differences in toxicity can be ascribed to variations in physicochemical properties and polymer system interactions. The lignin-containing nanocellulose is an interaction of extensive hydrogen bonds of the phenolic hydroxyl and carboxyl groups of lignin with the surface hydroxyl groups of nanocellulose. These polymer interactions were reported to exhibit enhanced antioxidant and antibacterial properties [109]. The hydrophobic nature of amorphous lignin can reduce the water sensitivity of polymers. The reduction in water sensitivity prevents the leaching of toxic residual monomers, oligomers, or additives into the surrounding ground matrices. This can explain the improved biocompatibility of the lignin-containing nanocellulose material. Native nanocellulose showed negligible cytotoxicity. However, cellulose nanofibrils of TEMPO-oxidized or phosphorylated types were shown to increase mitochondrial activity, pro-inflammatory cytokine production, and inflammation-related gene expression in human alveolar macrophages without outright cell death. This suggested potential inflammatory responses in lung tissues upon inhalation, and no reactive oxygen species was induced [109,170]. The literature is extremely limited on human in vivo studies, as most data are from in vitro models. It is worth noting that organosolv lignin, incorporated into hydrogels for tissue engineering, enhanced cell attachment without major toxic effects [171]. The differences in purity, molecular weight, sulfur content, and ash content of lignin can explain the cytotoxicity behavior of lignin in human cells.

## 9. Conclusions

The percentage crystallinity and cellulose content are principal selection criteria of agricultural waste biomass for the production of nanocellulose and nanofibrils, and including biochar-derived nanocarbons. Properties such as the composition, surface charge, surface area, zeta potential, morphology, and thermal decomposition temperatures can vary according to the diversity in crop types and extraction methods. Moreover, particle size, dimensions, and shape are some of the factors that can influence the end-user properties of nanocellulose. Pyrosis is the eminent strategy for the transformation of agricultural waste into biochar-derived nanoparticles, and yet there are limited studies on landfills into biochar for useful industrial applications.

### Emerging and Promising Future Prospective

The increasing global objectives on sustainable, biodegradable, and high-performance materials are driving the progress of research and development in cellulose-based nanoparticles by shifting from laboratory-scale concepts to industrial-scale application. Nevertheless, the cost of material extraction and production can be cost prohibitive. Future actions are seriously focused on reducing production costs, valorizing agricultural waste, and functionalizing surfaces for applications in electronics, biomedical engineering, and packaging. Beyond established uses, there is a need for the intensification of nanocellulose in smart devices (sensors), printable electronics, and environmental solutions like biodegradable packaging. It could also be integrated into the aerospace and automobile industries as lightweight composites, biomedicine for autonomous implants, and sustainability initiatives for circular economies.

Sustainable production: Develop low-cost green processes like biosynthesis, biocatalytic methods, and mechanochemical techniques; optimize existing methods (ionic liquids, deep eutectic solvents, enzymatic hydrolysis) to reduce costs; explore underutilized biomass sources such agricultural residues including bio-landfills for the production of biochar and graphene particles. Intensify the development of physical screening methods for the selection of agricultural waste biomass. These actions must be aimed at mass production for commercialization, responding to sustained supplies of raw materials in the industry.

Functionalization and modification: These are aimed at enhancing biocompatibility and minimizing structural damage. Develop sustainable techniques for composite molecular design and hybridization to produce nanomaterials with multifunctionality. Improve methods of characterization, aiming at key parameters that influence surface charge and surface chemistry.

Biomedical applications: Design optimized pH-responsive systems for drug delivery, tissue engineering scaffolds, and integrate Artificial Intelligence for smart implants. Develop comprehensive datasets for biocompatibility and toxicology, responding to compliance and standardization.

Energy and electronics: Develop cellulose-based hybrids for solar cells, supercapacitors, electrodes, and sensors; explore 3D printing and aerogel products for energy storage. Develop composites with improved electrical conductivity.

Composite materials: This is aimed at developing sustainable lightweight materials at scale, responding to the increasing demand for high-performance composite polymers in automotives, aerospace, and construction.

## Figures and Tables

**Table 1:** Cellulose, lignin and hemicellulose contents of agricultural waste biomass.

Fibrous Biomass	Cellulose	Lignin	Hemicellulose	Treatment/Extraction Process	Reference
Rice husk	30–70	3–25	10–20	Alkaline, bleaching agent, acid hydrolysis	Hafid, H.S. et al. [7]
Rice husk	50	27	10	NaOH treatment, water washing	Ponce, J. et al. [8]
Rice husk	22–57	-	-	Acetic acid, bleaching (NaOH/H_2_O_2_)	Vu, A.N. et al. [9]
Rice husk	16	21	36	Removal of dust, without treatments	Lin, L. et al. [10]
Rice straw	18	14	47	Removal of dust, without treatments	Lin, L. et al. [10]
Sugarcane bagasse	58	12	17	NaOH treatment, water washing	Ponce, J. et al. [8]
Sugarcane bagasse	39	26	24	Acid hydrolysis	Rodríguez-Chong, A. et al. [11]
Sugarcane bagasse	26–47	19–23	14–23	Alkaline treatment, hot water wash	Mahmud, M.A. and Anannya, F.R. [12]
Sugarcane bagasse	41	23	21	High temperature alkaline treatment	Song, G. et al. [13]
Peanut shell	36–59	27	7	Deep eutectic solvents	Lu, A. et al. [14]
Peanut shell	32–34	32	18	Standard and extraction methods	Husna, M. and Vasantharuba, S. [15]
Peanut shell	32–37	27–30	7–9	Inoculum of culture incubation process	Anike, F. et al. [16]
Peanut shell	45	36	6	-	Pączkowski, P. et al. [17]
Corn husk	55	9	27	NaOH treatment, water washing	Ponce, J. et al. [8]
Corn stalk	38–44	5–10	17–27	Inoculum of culture incubation process	Anike, F. et al. [16]
Corn bract	36	39	15	Enzymatic hydrolysis without pre-treatment	Anike, F. et al. [16]
Corn bract	52	35	4	Acetic acid, enzymatic hydrolysis	Anike, F. et al. [16]
Corn bract	76	13	2	Ammonium, enzymatic hydrolysis	Anike, F. et al. [16]
Olive leaves	6–9	40	4–9	Acid hydrolysis	Garcia-Maraver, A. et al. [18]
Olive pruning	20	27	10–11	Acid hydrolysis	Garcia-Maraver, A. et al. [18]
Olive wood	31–32	24	11–15	Acid hydrolysis	Garcia-Maraver, A. et al. [18]
Pineapple leaf	30	22	37	NaOH and sodium chlorite treatment	Mansora, A.M. et al. [19]
Pineapple leaf	66	4	20	Bleaching agent	Daud, Z. et al. [20]
Pineapple stem	37	20	34	NaOH and sodium chlorite treatment	Mansora, A.M. et al. [19]
Pineapple root	42	19	32	NaOH and sodium chlorite treatment	Mansora, A.M. et al. [19]
Banana stem	35	12	25	NaOH and sodium chlorite treatment	Mansora, A.M. et al. [19]
Banana stem (outer)	40	13	25	NaOH and sodium chlorite treatment	Mansora, A.M. et al. [19]
Cassava peel	38	8	37	Dust removal, without treatment	Aripin, A.M. et al. [21]
Cassava peel	40	12	21	Dust removal, without treatment	Aripin, A.M. et al. [21]
Cassava peel	38	8	37	Bleaching agent	Daud, Z. et al. [20]

**Table 2:** Production of nanoparticles from lignocellulose and cellulose.

Nanomaterial	Material	Method	Activation Process	Surface Effect	Reference
Graphene	Rice husk	Carbonization Pyrosis 400 °C	Mixture of biochar with KOH in 1:2 ratio, annealing at 800 °C, washing and drying	Few-layered graphene with agglomeration of silica particles (porous)	Mubarik, S. et al. [54]
Graphene	Peanut shells	Carbonization Pyrosis 200 °C	Mixture of biochar and FeCl_3_·6H_2_O in water. Adjust pH HCl (98% Betaine Hydrochloride). Washing and drying at 100 °C, milling	Two-dimensional (2D) material, network of SP^2^-bonded carbon atoms	Aro-Modiu, O. et al. [55]
Graphene oxide	Graphene	-	Mixture of graphene powder, sodium nitrate and H_2_SO_4_ in ice bath. Adding KMnO_4_. Reaction termination with H_2_O_2_. Washing, filtration. Drying 30 °C	Polar groups (surface HO^−^ groups), surface area ≈ 2630 m^2^ g^−1^	Aro-Modiu, O. et al. [55] Liu, J. et al. [56]
Graphene	Rubber seed shell	Pyrolysis 700 °C for 90 min	Mixture of biochar to KOH in 1:3 ratio under N_2_ at different temperatures (600, 700, 800 and 900 °C), 3 h	Surface area 712 m^2^g^−1^	Anthonysamy, S. et al. [57]
Cellulose nanocrystals	Peanut shells	lignocellulose	Peanut shell powder (500-μm sieve), hot rinse and dry. Treatment: HCl, NaOH. Precipitate washed, centrifuged. Residual fibers sonicated and dried. Fibers hydrolyzed with H_2_SO_4_, cold water wash, centrifuge, ultrasonication, drying	High antifungal and antibacterial activity. Positive reaction and sensitivity against Gram (−) and Gram (+) strains of pathogens	Terea, H. et al. [58]
Cellulose nanocrystal	Tea leaf waste fiber	Cellulose extraction	Acid hydrolysis: cellulose treated with H_2_SO_4_ (pre-heated). Processes of dialysis of suspension and ultrasonic.	-	Abdul Rahman, N.H. et al. [59]
Nanocellulose crystals	Tea stalk	Cellulose isolation	Acid hydrolysis	Lower zeta (ζ) potential −33 mV	Guo, Y. et al. [60]
Nanoparticles	Cassava peel	Starch extraction	Sulfuric acid hydrolysis, washing and drying		Abdul Rahman, N.H. et al. [59]
Silica nanoparticles	Sugarcane bagasse	Extraction, precipitation	Acid hydrolysis, alkali hydrolysis	Surface area 111 m^2^g^−1^	Mohd, N.K. et al. [61]
Cellulose nanofibers	Banana peel		Alkaline hydrolysis [62], bleaching (NaClO_2_), acid hydrolysis (H_2_SO_4_) Mechanical process: two-stage high-pressure homogenizer	Between −16 mV and −44 mV	Pelissari, F.M. et al. [63]

**Table 3:** Different techniques and types of ball milling.

Type of Ball Mills	Source & Specification	Process Input	Sample	Primary Properties	Reference
Planetary Ball Mill	PM100; Retsch Corporation(Retsch-Allee, Germany), stainless steel jar (500 mL), stainless steel balls 2.4 mm in diameter × 800 balls	settings at 5 min ON and 5 min OFF Milling speed of 510–630 rpm	Pinewood biochar, initial size of around 3 mm	Fine powder 212–453 nm Zeta potential (mV) −31.3 ± 2.6	Naghdi, M. et al. [70]
Planetary ball mill	ITO LP-1. 80 mL jar with different ball diameters of 10, 5, and 2 mm × 5, 4, and 3 balls, respectively	speed of 300 rpm, time treatment 0.5, 1, 2, and 3 h, acid hydrolysis-assisted	Grade 3 mm Chr cellulose 38 µm (400 mesh) size of cellulose powder	Yield 77–90%, native cellulose type I, size 3–13 nm, maximum decomposition temperature 300–330 °C with mass loss 75 wt%	Phanthong, P. et al. [71]
Tumbler Ball Mill	MA500, Marconi, with alumina balls. Jar 1 L 70% of balls and 30% samples	speed 200 rpm, Treatment times: 1 h, 2 h, 3 h, and 4 h. ethanol-ultrasonic-assisted	Cellulose extracted from eucalyptus sawdust using NaClO_2_, NaOH and KOH	Yield ~80 wt%. Zeta potential (mV) −24 to −60	Ferreira, R.R. et al. [72]
Planetary ball mill	ITO LP-1 Planetary pot mill,	milling speed of 400 rpm for 2 h	Cellulose powder	Crystal size 2–4 nm, degradation 220–410 °C with thermal decomposition peak at 373 °C with 80 wt% loss	Phanthong, P. et al. [73]
Planetary ball mill	XQM-0.4A, Tencan powder (Changsha, China)	speed of 400 rpm, 3 h in cycles of 20 min milling per 10 min rest. NaOH-assisted.	Cellulose from pineapple peel	Nanofibrils size 19–24 nm, Zeta potential (mV) −22 to −28.	Wang, Y. et al. [74]
Planetary ball mill	Naraya-MPM-2 × 250H mill (Amin Asis Fanavar Pars), 250 mL jar, 60% stainless steel balls of 0.5–2 cm, 30% sample	speed 200 rpm for 2 h. dispersed in deionized water prior ultrasonic-assisted	Cellulose from Cuminum cyminum waste	Zeta potential (mV) −25. Size 25 nm. Degradation peak around 330 °C	Hoseinpour, Z. et al. [75]
Tumbler Ball Mill	impact ball mill (MA500, Marconi Ltda., Piracicaba, SP, Brazil)	alumina balls (diameter of 21 mm), 70 g of alumina balls for 1 g of cellulose fiber. Time 6, 9, and 12 h. Ethanol-assisted	Cellulose from corn stalks, cobs, and husks	Zeta potential (mV) −15 to −41 (stalk), −13 to −42 (husk), −30 to −38 (comb). Size 70–195 nm	dos Santos, D.F. et al. [76]

**Table 4:** Nanoparticle dimensions.

Dimension	Nanoscale	Shape	Nanomaterials	Reference
Zero dimension (0D)	Dimensions length, breadth, height (x,y,z) < 100 nm	Spherical, quasi-sphere, cubic, polygonal	Carbon dots, fullerene, clusters, grains, nanoparticles	Paras et al. [96]
One-dimensional (1D)	Two dimensions (x,y) < 100 nm, and third dimension (z) > 100 nm	Needle-like	Linear structures, carbon nanotubes, metals or metal oxide nanowires, polymer nanowires, nanofibers, hybrid materials	Su, B. et al. [97]
Two-dimensional (2D)	One dimension (x) < 100 nm, other two dimensions (y,z) undefined	Atomically thin sheets, sheet-like honeycomb	Nanofilms, nanolayers, and nanocoatings	Li, X. and Wang, J. [98]
Three-dimensional (3D)	Dimensions (x,y,z) > 100 nm, not confined to the nanoscale	Nano-cubes, fullerenes, dendrimers, and nanocages	Cellulose nanocrystals (CNCs) and nanofibrils (CNFs)	Dutta, S. et al. [99]

**Table 5:** Crystallinity of nanocellulose.

Nanoparticles	Source of Waste Biomass	Technique	Crystalline Properties	Reference
Cellulose nanofibrils	Pineapple peel	XRD (X’Pert3 Powder, Malvern Panalytical, Almelo, The Netherlands)	Crl 38–44%, Crystal size (D) 2–3 nm	Wang, Y. et al. [74]
Nanocellulose	Cumin husk	XRD, Karlsruhe instrument (Karlsruhe, Germany), input: CuKα radiation (λ = 0.1542 nm, 40 kV, 40 mA). Position: 2θ range of 5–65°, scan rate of 3°/min	XRD peaks at 2θ = 18° and 22.6° Clr 69.3% at 2θ = 22.6° Crystal size 3.76 nm	Hoseinpour, Z. et al. [75]
Nanocellulose	Maguey fiber	XRD, LabX-6000, SHIMADZU, Kyoto, Japan. Input: 40.0 kV and 30.0 mA with Cu Kα radiation (1.5148 Å). 2θ range of 2–70°, scan speed 1°/min	XRD peaks ~2θ = 15.52° and 22.64°, Crl 74.80% at 2θ = 22.64°	Sumarago, E.C. et al. [120]
Raw fiber	Keya leaf (alkali and bleach treatment)	Wider angle XRD, BRUKER D8 ADVANCE, input: Cu Kα radiation (α = 0.154 nm) at 40 mA and 50 kV	Raw fiber Crl 45.35% at 2θ = 14.79°, 22.59°, 24.25°, and 29.97. Alkali fiber Crl 54.69% at 2θ = 22.97° Bleached fiber Crl 43.43% at 2θ = 22.8°	Hossain, M.I. et al. [121]
Nanocellulose crystals	Keya leaf	Wider angle XRD, BRUKER D8 ADVANCE, Ettlingen, Germany, input: Cu Kα radiation (α = 0.154 nm) at 40 mA and 50 kV	Clr 61.31% at 2θ = 19.9° and 22.44°	Hossain, M.I. et al. [121]
Nanocellulose crystals	Peanut shells	XRD, RIGAKU MINIFLEX-600 input: Cu Kα (λ = 1.54 A0), scanning in a 2θ range of 10–50°	Crl 26–66% at 2θ = 22.6°	Punnadiyil, R.K. et al. [122]
Nanocrystalline cellulose	Banana fiber	XRD, Shimadzu XRD-6000. Input: voltage of 30 kV and current of 30 mA, 2θ = 5–60°, scan rate 2 °C/min	XRD peaks at 2θ = 16.1°, 22.8° and 34.9° Crl 62% at 2θ = 19 and 22.2°	Mishra, S. et al. [123]
Nanocellulose	Rice husks (variety)	XRD, MiniFlex 600 (Rigaku, Tokyo, Japan). Input: Cu Kα λ = 1.54, acceleration potential =40 kV, current = 15 mA. Scan range of 2θ = 2–60°, scan rate 3°/min	XRD peaks 2θ = 16.3°, 22.4° and 34.5° for raw husk. Crl for husk 40–56% at 2θ = 22.4° Crl for cellulose 46–66% at 2θ = 22.4° Crl for nanocellulose 59–77% at 2θ = 22.4°	Rashid, S. and Dutta, H. [119]

Crl = Crystallinity, 2θ = theta position.

**Table 6:** Thermal properties of nanomaterial.

Nanoparticles	Source of Biomass	Technique	Thermal Properties	Reference
Cellulose	Eucalyptus saw dust	TGA, heat 20–600 °C. instrument: STA 6000 (PerkinElmer, USA)	T_max_ 367 °C	Ferreira, R.R. et al. [72]
Nanocellulose	Eucalyptus saw dust	TGA, heat 20–600 °C. instrument: STA 6000 (PerkinElmer, USA)	T_maz_ 321–342°	Ferreira, R.R. et al. [72]
Nanocellulose	Cumin husk	TGA, 20–600 °C, heat rate 20 °C/min, STA 6000 (PerkinElmer, USA)	initial weight loss (50–150 °C), T_max_ (275–365 °C), thermal decomposition (>400 °C)	Hoseinpour, Z. et al. [75]
Extracted fiber	Bamboo	Thermal analyzer, temp 40–700 °C, Hitachi STA 7300 (Hitachi, Tokyo, Japan), heating rate of 10 °C/min. Sample weight (3–5 mg).	minor weight 5–7% (100–300 °C), major weight loss 40–41% at peak decomposition (300–400 °C), final weight loss 10–12% at final decomposition (>350 °C)	Verma, Y.K. et al. [126]
Nanocellulose	Bamboo	Thermal analyzer, temp 40–700 °C, Hitachi STA 7300, heating rate of 10 °C/min. Sample weight (3–5 mg).	minor weight 5–6% (100–300 °C), major weight loss 40–41% at peak decomposition (300–400 °C), final weight loss 10–15% at final decomposition (>350 °C)	Verma, Y.K. et al. [126]
Cellulose	Cellulose paper	TGA, thermal analyzer (DTG-60H, Shimadzu), temp 20–600 °C, heating rate 10 °C/min	major degradation temp (370 °C), weight loss (87%) and residue weight loss (6%) at 600 °C	Phanthong, P. et al. [73]
Nanocellulose	Cellulose paper	TGA, thermal analyzer (DTG-60H, Shimadzu), temp 20–600 °C, heating rate 10 °C/min	major degradation temp (200–420 °C), weight loss (75%), max decomposition temp (340–350 °C). residue weight loss (15%) at 600 °C	Phanthong, P. et al. [73]
Fibrous	Pineapple pee (hot, bleach and alkali treated)	TGA, thermal analyzer (TGA550, TA Instruments, New Castle, DE, USA), temp 30–600 °C, heating rate 10 °C/min	onset temp (220–240 °C) and max temp (340–345 °C), char residue (5–19%)	Wang, Y. et al. [74]
Cellulose nanofibrils	Pineapple peel	TGA, thermal analyzer (TGA550, TA Instruments, New Castle, DE, USA), temp 30–600 °C, heating rate 10 °C/min	onset temp (230–245 °C) and max temp (316–330 °C), char residue (14–17%)	Wang, Y. et al. [74]

## Data Availability

No new data were created or analyzed in this study. Data sharing is not applicable to this article.
